# Psychopharmacological treatment of anxiety and depression in patients with liver and/or kidney disease. Pharmacodynamics and pharmacokinetic considerations, drug interactions.

**DOI:** 10.1192/j.eurpsy.2025.176

**Published:** 2025-08-26

**Authors:** M. Stuhec

**Affiliations:** 1 Pharmacology Department, Medical Faculty Maribor, University of Maribor, Maribor; 2 Clinical Pharmacy, Ormoz Psychiatric Hospital, Ormoz, Slovenia

## Abstract

**Abstract:**

Psychopharmacological treatment of anxiety and depression in patients with liver and/or kidney disease is complex, primarily due to the differences in pharmacokinetics compared to healthy individuals. Most medications are excreted via the kidney and/or liver, so dose adjustments are often necessary in these patients. Drug-drug interactions are also a significant clinical concern in liver and/or kidney disease, making regular monitoring essential. These factors may contribute to treatment relapse and severe adverse drug reactions associated with psychotropic medications and comedications.

Medication selection is, therefore, based on these considerations. Clinicians must regularly monitor kidney and liver function, although there are no clear global recommendations. A summary of product characteristics is the primary guideline for dose adjustments related to kidney and liver function, though it has limitations and is updated slowly. The most important monitoring parameters for these patients are liver enzymes and serum creatinine levels.

Patients with liver and/or kidney disease are often excluded from randomized controlled trials, meta-analyses, and clinical guidelines, resulting in a lack of data specific to this population. This issue is even more relevant in elderly patients treated with polypharmacy, as many somatic medications can worsen liver and/or kidney function in patients with anxiety and depression. In this context, some medications, such as vortioxetine, trazodone, agomelatine, quetiapine, and sertraline, are less affected by kidney disease in terms of pharmacokinetics.

Several tools are available for prudent medication selection and monitoring in these patients, including medication lists (e.g., Beers, Priscus), therapeutic drug monitoring (TDM), and collaboration with clinical pharmacists and/or pharmacologists. In this presentation, the presenter will discuss this topic from both pharmacological and clinical perspectives. Participants will learn the fundamental pharmacokinetic aspects necessary for medication selection in these patients, which are valuable for daily practice.

**Disclosure of Interest:**

None Declared

